# The Regulation of Exosporium-Related Genes in *Bacillus thuringiensis*

**DOI:** 10.1038/srep19005

**Published:** 2016-01-25

**Authors:** Qi Peng, Guiwei Kao, Ning Qu, Jie Zhang, Jie Li, Fuping Song

**Affiliations:** 1 State Key Laboratory for Biology of Plant Diseases and Insect Pests, Institute of Plant Protection, Chinese Academy of Agricultural Sciences, Beijing; 2 College of Life Sciences, Northeast Agriculture University, Harbin, China

## Abstract

*Bacillus anthracis, Bacillus cereus*, and *Bacillus thuringiensis* (Bt) are spore-forming members of the *Bacillus cereus* group. Spores of *B. cereus* group species are encircled by exosporium, which is composed of an external hair-like nap and a paracrystalline basal layer. Despite the extensive studies on the structure of the exosporium-related proteins, little is known about the transcription and regulation of exosporium gene expression in the *B. cereus* group. Herein, we studied the regulation of several exosporium-related genes in Bt. A SigK consensus sequence is present upstream of genes encoding hair-like nap proteins (*bclA* and *bclB*), basal layer proteins (*bxpA, bxpB, cotB*, and *exsY *), and inosine hydrolase (*iunH*). Mutation of *sigK* decreased the transcriptional activities of all these genes, indicating that the transcription of these genes is controlled by SigK. Furthermore, mutation of *gerE* decreased the transcriptional activities of *bclB, bxpB, cotB*, and *iunH* but increased the expression of *bxpA*, and GerE binds to the promoters of *bclB, bxpB, cotB, bxpA*, and *iunH*. These results suggest that GerE directly regulates the transcription of these genes, increasing the expression of *bclB, bxpB, cotB*, and *iunH* and decreasing that of *bxpA*. These findings provide insight into the exosporium assembly process at the transcriptional level.

*Bacillus anthracis, Bacillus cereus*, and *Bacillus thuringiensis* (Bt) are spore-forming members of the *Bacillus cereus* group^1^. These species vary in terms of host range and virulence^2^ and are mainly distinguished by the genes contained in their plasmids. Bt forms parasporal crystals during the stationary phase of growth; these crystals are toxic to a wide variety of insect larvae^3^, making Bt strains the most commonly used biological pesticide worldwide.

The genus *Bacillus* encompasses species capable of forming highly resistant dormant endospores as a response to harsh environmental conditions. Spores of the *B. cereus* group are complex, multilayered structures. The nucleoid-containing core is enclosed within a peptidoglycan cortex, which is surrounded by the spore coat^4^. Spores of all the *B. cereus* group species are encircled by an additional loose-fitting layer called the exosporium^5^, which is not present on other species such as *Bacillus subtilis*, for which the coat constitutes the outermost layer of the mature spore^6^. The exosporium is a balloon-like layer that acts as the outer permeability barrier of the spore and contributes to spore survival and virulence^7^. The exosporium also interacts with host cells during infection^8^.

Many characteristics of the exosporium have been previously elucidated. The exosporium is separated from the spore coat by a region known as the interspace and is the final layer of the spore to be assembled^9^,^10^,^11^,^12^. It is composed of an external hair-like nap and a paracrystalline basal layer and contains approximately 20 different proteins^13^,^14^,^15^, which are deposited around the spore in a progressive encasement process^9^,^10^,^11^. The assembly of the nap closely follows the progressive assembly of the basal layer^9^,^11^. The filaments of the nap are formed by trimers of the collagen-like glycoprotein BclA, which is involved in early interactions with the host surface^16^. BclA is attached to the underlying basal layer by its N-terminal domain^9^, which is followed by an extensively glycosylated collagen-like central region^17^ and a C-terminal globular β-jellyroll domain that promotes trimer formation^16^,^18^. A second collagen-like protein, BclB, is also present in the exosporium. BclB possesses an N-terminal sequence that targets it to the exosporium and is similar in sequence to a cognate-targeting region in BclA^19^. The attachment of nearly all BclA trimers requires the basal layer protein BxpB^14^, which has been implicated as a foundation upon which nap proteins are assembled. BclA and BxpB form high molecular mass complexes, which are stable under conditions that normally disrupt non-covalent interactions and disulfide bonds^10^,^20^. However, BclB lacks sequence similarity to the region of BclA thought to mediate attachment to the basal layer via covalent interactions with BxpB^19^. In addition, several proteins have been implicated in exosporium formation, including BxpA^13^, CotB^15^, CotY^21^, ExsA^22^, ExsB^23^, ExsK^24^, ExsFB^11^,^20^, ExsM^25^, and ExsY^21^,^26^,^27^. Enzymes associated with the exosporium, including alanine racemase^27^, inosine hydrolase^15^, and superoxide dismutase^13^, may be involved in preventing premature germination and providing protection against macrophages by detoxifying superoxide free radicals^28^,^29^.

Despite the extensive studies on the structure of the exosporium-related proteins, little is known about the transcription and regulation of exosporium gene expression in the *B. cereus* group. Herein, we demonstrate that the transcription of *bclA, bclB, bxpA, bxpB, cotB, exsY*, and *iunH* are controlled by RNA polymerase sigma factor SigK in Bt HD73. Furthermore, the expression of *bclB, bxpA, bxpB, cotB*, and *iunH* is directly regulated by GerE. *gerE* encodes the terminal transcription factor in the sporulation regulatory cascade in *Bacillus subtilis*. GerE is a small DNA-binding protein that is both an activator and a repressor in the mother cell that regulates the transcription of many genes involved in spore coat synthesis and assembly in the late stages of sporulation and germination^30^,^31^,^32^. GerE acts in conjunction with SigK-containing RNA polymerase to turn on the expression of the final class of sporulation genes. The appearance of GerE also switches off the expression of some genes that had been activated by SigK^31^.

## Results

### Transcriptional activity of hair-like nap protein genes

**Figure 1:**
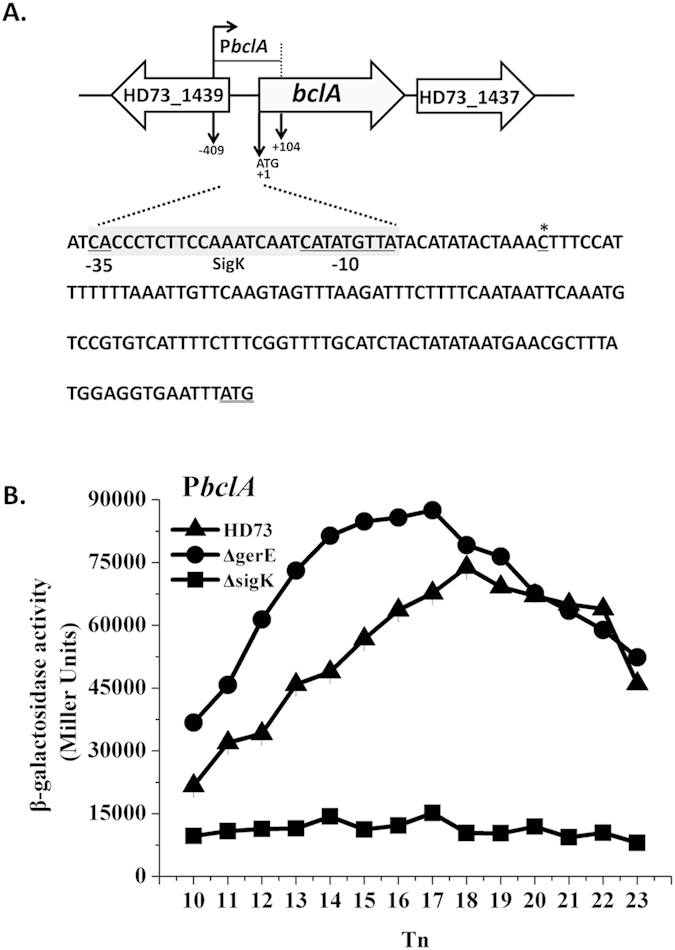
Nucleotide sequence and transcriptional activity of the *bclA* promoter. (**A**) Nucleotide sequence analysis. The indicated promoter region, 409 bp upstream and 104 bp downstream of the start codon (double-underlined), was fused with *lacZ*. Transcriptional start site (TSS, indicated by asterisks) is located 120 bp upstream from the start codon of the *bclA* gene. The SigK consensus sequence is indicated with a gray box, and the putative -35 and -10 sequences are underlined. (**B**) β-galactosidase activity assay of P*bclA* in wild-type HD73 (▲), *sigK* mutant (◼), and *gerE* mutant (●). T_0_ is the end of the exponential phase, and T_n_ is n hours after T_0_. Values represent mean of at least three independent replicates; error bars represent standard deviation.

**Figure 2:**
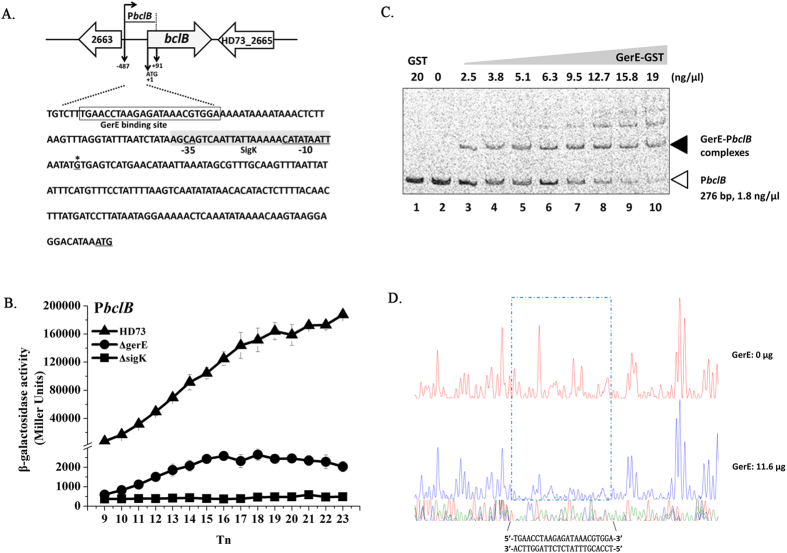
Nucleotide sequence and transcriptional activity of the *bclB* promoter. (**A**) Nucleotide sequence analysis. The indicated promoter region, 487 bp upstream and 91 bp downstream of the start codon (double-underlined), was fused with *lacZ*. Transcriptional start site (TSS, indicated by asterisks) is located 150 bp upstream from the start codon of the *bclB* gene. The SigK consensus sequence is indicated, and the putative -35 and -10 sequences are underlined with a gray box. The GerE binding site is boxed. (**B**) β-galactosidase activity assay of P*bclB* in wild-type HD73 (▲), *sigK* mutant (◼), and *gerE* mutant (●). T_0_ is the end of the exponential phase, and Tn is n hours after T_0_. Values represent mean of at least three independent replicates; error bars represent standard deviation. (**C**) Electrophoresis mobility shift assay of the *bclB* promoter fragment (276 bp) after incubation with GerE. Lane 1, FAM-labeled P*bclB* probe incubated with GST protein; lane 2, FAM-labeled P*bclB* probe; lanes 3–10, incubation of the probe with increasing concentrations of purified GerE indicated at the top of the figure. (**D**) Protection of a 23-bp sequence in the *bclB* promoter by GerE, as revealed by DNase I footprinting protection assay. The fluorograms correspond to the DNA in the protection reactions (with 0 and 11.6 μg GerE).

We identified 17 exosporium homologous genes with known functions in *B. cereus* and *B. anthracis* in Bt HD73 (Table 1) comprising genes encoding the hair-like nap proteins, basal layer proteins, and enzymes. A major component of the hair-like nap is the glycosylated collagen-like protein BclA. A second collagen-like protein, BclB, is also present in the exosporium^19^. In Bt HD73, *HD73_1438* (*bclA*) and *HD73_2664* (*bclB*) encode BclA and BclB and have 67.8% and 90.0% identity, respecively, to homologous genes in *B. anthracis* Sterne strain 7702^33^ and *B. cereus* ATCC 10876^34^. To determine the transcription start site (TSS) of *bclA* and *bclB*, 5′-RACE analysis was performed as described in the Methods. The TSSs of *bclA* and *bclB* were confirmed to be a single 5′-end nucleotide residue C and G located 120 bp and 150 bp upstream of the start codon according to the sequences of 20 random clones, respectively (Figs 1A and 2A). Analysis of the *bclA* and *bclB* promoter sequences identified sequences CAC(-N_16_-)CATATGTTA and AGC(-N_16_-)CATATAATT upstream of the *bclA* and *bclB* TSS, respectively, which are similar to the consensus sequences recognized by SigK-containing RNA polymerase^35^, with the putative binding site centered at -10 and -35 nt with appropriate spacing (16 nt) between these consensus sequences (Figs 1A and 2A). SigK is a sigma factor that plays a role in the late stage of sporulation, and some SigK-dependent genes are negatively or positively regulated by GerE in the late stage of sporulation^31^. Thus, to study the transcription and regulation of the promoters P*bclA* and P*bclB*, P*bclA-lacZ* and P*bclB-lacZ* fusions were constructed and transformed into Bt wild-type strain HD73 and mutant strains, HD(Δ*sigK*) and HD(Δ*gerE*). The β-galactosidase assay showed that the transcriptional activity of P*bclA* was sharply decreased from T_10_ to T_23_ in HD(Δ*sigK*) (Fig. 1B). It was slightly increased from T_10_ to T_18_ in HD(Δ*gerE*), and with no significant difference from T_18_ to T_23_ compared with that of wild-type strain HD73 (Fig. 1B). However, the transcriptional activity of P*bclB* was sharply decreased from T_9_ to T_23_ both in HD(Δ*sigK*) and HD(Δ*gerE*) compared with that of HD73 (Fig. 2B). To determine whether GerE directly or indirectly regulates the P*bclA* and P*bclB*, GerE-GST protein was expressed in *E. coli* and purified. The ability of GerE to bind to a DNA fragment containing the P*bclA* (267 bp) and P*bclB* (276 bp) promoters was examined by EMSA. FAM-labeled fragments containing the promoter regions of *bclB* were incubated with different amounts of GerE and assayed for the formation of protein-DNA complexes. Slower-migrating probe-protein complexes were observed upon incubation with increasing amounts of GerE (Fig. 2C). It indicated that GerE recognizes and specifically binds to sequences within the *bclB* promoter fragment. To precisely determine the GerE-binding site in the *bclB* promoter, DNase I footprinting assays were carried out using the same *bclB* promoter fragment used in the EMSA (Fig. 2D). A 23-bp fragment corresponding to the boxed sequence in the *bclB* promoter region (Fig. 2A) was protected by GerE binding. In sharp contrast, GerE did not bind to labeled *bclA* promoter (Additional file 1). This may result from the lack of direct binding, from a purified GerE protein partially defective in binding or from unfavorable *in vitro* binding conditions. These results indicated that transcription of P*bclA* and P*bclB* are controlled by SigK in the late stage of sporulation and that P*bclB* is directly activated by GerE, while P*bclA* is negatively regulated by GerE.

### Transcriptional activity of basal layer protein genes

**Figure 3:**
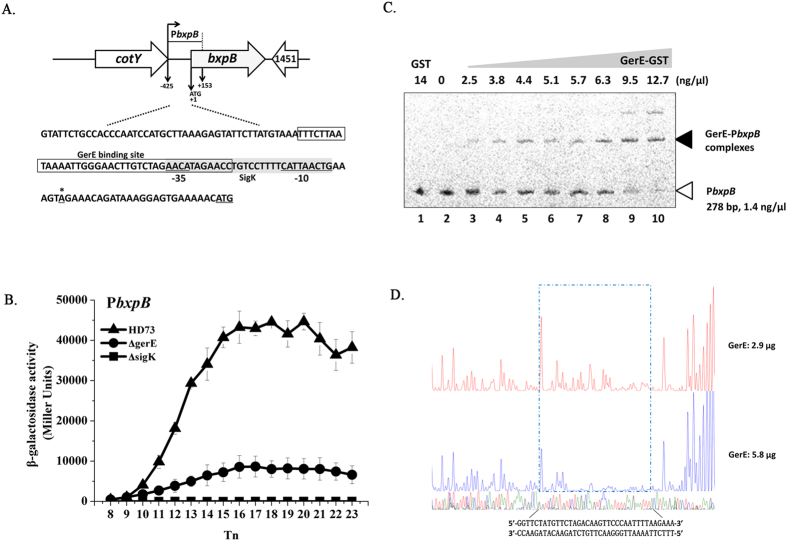
Nucleotide sequence and transcriptional activity of the *bxpB* promoter. (**A**) Nucleotide sequence analysis. The indicated promoter region, 425 bp upstream and 153 bp downstream of the start codon (double-underlined), was fused with *lacZ*. Transcriptional start site (TSS, indicated by asterisks) is located 24 bp upstream from the start codon of the *bxpB* gene. The SigK consensus sequence is indicated, and the putative −35 and −10 sequences are underlined with a gray box. The GerE binding site is boxed. (**B**) β-galactosidase activity assay of P*bxpB* in wild-type HD73 (▲), *sigK* mutant (◼), and *gerE* mutant (●). T_0_ is the end of the exponential phase, and Tn is n hours after T_0_. Values represent mean of at least three independent replicates; error bars represent standard deviation. (**C**) Electrophoresis mobility shift assay of the *bxpB* promoter fragment (278 bp) after incubation with GerE. Lane 1, FAM-labeled P*bxpB* probe incubated with GST protein; lane 2, FAM-labeled P*bxpB* probe; lanes 3–10, incubation of the probe with increasing concentrations of purified GerE indicated at the top of the figure. (**D**) Protection of a 37-bp sequence in the *bxpB* promoter by GerE, as revealed by DNase I footprinting protection assay. The fluorograms correspond to the DNA in the protection reactions (with 2.9 and 5.8 μg GerE).

**Figure 4:**
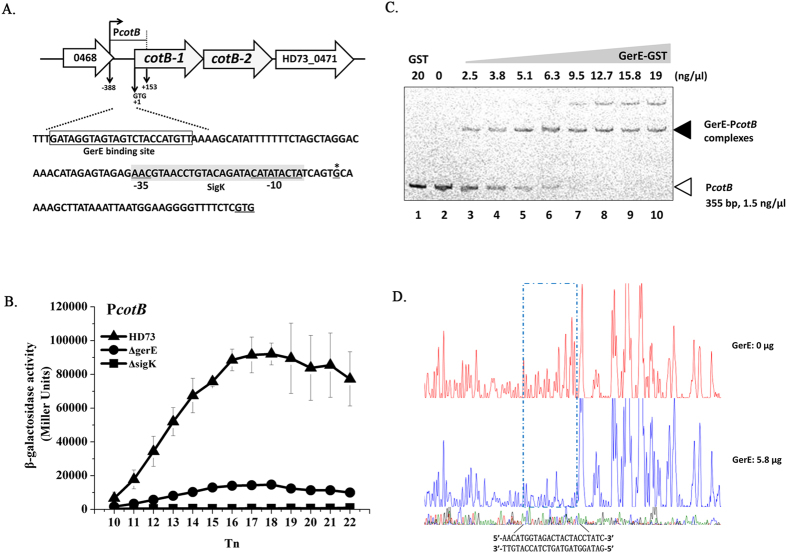
Nucleotide sequence and transcriptional activity of the *cotB* promoter. (**A**) Nucleotide sequence analysis. The indicated promoter region, 388 bp upstream and 153 bp downstream of the start codon (double-underlined), was fused with *lacZ*. Transcriptional start site (TSS, indicated by asterisks) is located 33 bp upstream from the start codon of the *cotB* gene. The SigK consensus sequence is indicated, and the putative −35 and −10 sequences are underlined with a gray box. The GerE binding site is boxed. (**B**) β-galactosidase activity assay of P*cotB* in wild-type HD73 (▲), *sigK* mutant (◼), and *gerE* mutant (●). T_0_ is the end of the exponential phase, and Tn is n hours after T_0_. Values represent mean of at least three independent replicates; error bars represent standard deviation. (**C**) Electrophoresis mobility shift assay of the *cotB* promoter fragment (355 bp) after incubation with GerE. Lane 1, FAM-labeled P*cotB* probe incubated with GST protein; lane 2, FAM-labeled P*cotB* probe; lanes 3–10, incubation of the probe with increasing concentrations of purified GerE indicated at the top of the figure. (**D**) Protection of a 33-bp sequence in the *cotB* promoter by GerE, as revealed by DNase I footprinting protection assay. The fluorograms correspond to the DNA in the protection reactions (with 0 and 5.8 μg GerE).

**Figure 5:**
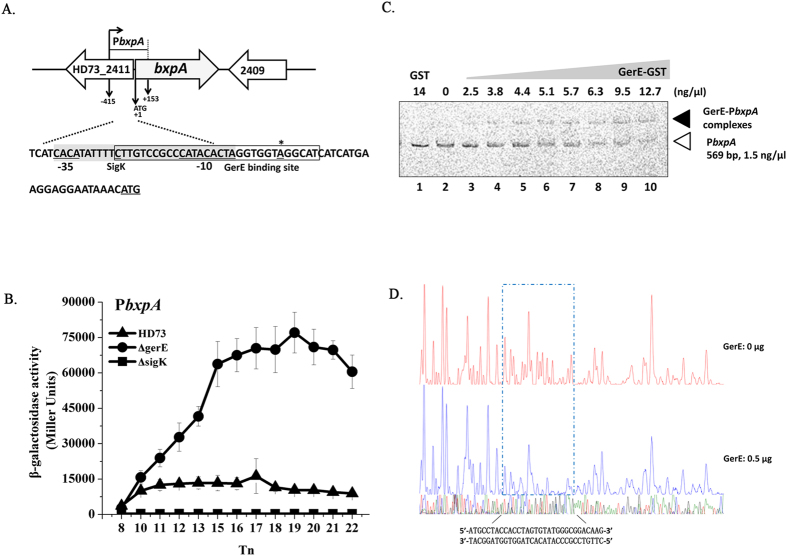
Nucleotide sequence and transcriptional activity of the *bxpA* promoter. (**A**) Nucleotide sequence analysis. The indicated promoter region, 415 bp upstream and 153 bp downstream of the start codon (double-underlined), was fused with *lacZ*. Transcriptional start site (TSS, indicated by asterisks) is located 26 bp upstream from the start codon of the *bxpA* gene. The SigK consensus sequence is indicated, and the putative −35 and −10 sequences are underlined with a gray box. The GerE binding site is boxed. (**B**) β-galactosidase activity assay of P*bxpA* in wild-type HD73 (▲), *sigK* mutant (◼), and *gerE* mutant (●). T_0_ is the end of the exponential phase, and Tn is n hours after T_0_. Values represent mean of at least three independent replicates; error bars represent standard deviation. (**C**) Electrophoresis mobility shift assay of the *bxpA* promoter fragment (569 bp) after incubation with GerE. Lane 1, FAM-labeled P*bxpA* probe incubated with GST protein; lane 2, FAM-labeled P*bxpA* probe; lanes 3–10, incubation of the probe with increasing concentrations of purified GerE indicated at the top of the figure. (**D**) Protection of a 31-bp sequence in the *bxpA* promoter by GerE, as revealed by DNase I footprinting protection assay. The fluorograms correspond to the DNA in the protection reactions (with 0 and 0.5 μg GerE).

**Figure 6:**
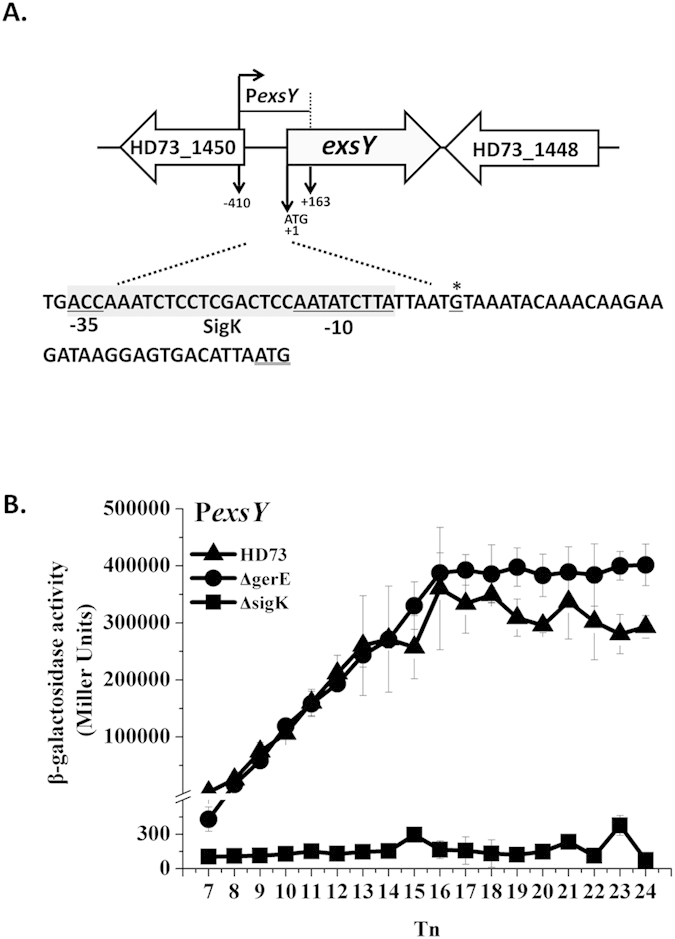
Nucleotide sequence and transcriptional activity of the *exsY* promoter. (**A**) Nucleotide sequence analysis. The indicated promoter region, 410 bp upstream and 163 bp downstream of the start codon (double-underlined), was fused with *lacZ*. Transcriptional start site (TSS, indicated by asterisks) is located 33 bp upstream from the start codon of the *exsY* gene. The SigK consensus sequence is indicated with a gray box, and the putative −35 and −10 sequences are underlined. (**B**) β-galactosidase activity assay of P*exsY* in wild-type HD73 (▲), *sigK* mutant (◼), and *gerE* mutant (●). T_0_ is the end of the exponential phase, and T_n_ is n hours after T_0_. Values represent mean of at least three independent replicates; error bars represent standard deviation.

We studied the transcription and regulation of four basal layer protein genes *bxpA* (HD73_2410), *bxpB* (HD73_1452), *cotB* (HD73_0469), and *exsY* (HD73_1449). These genes have 75.4%, 97.0%, 76.9%, and 87.0% identity, respectively, to homologous genes in *B. anthracis* or *B. cereus* (Table 1). The TSSs of *bxpA, bxpB, cotB*, and *exsY* were confirmed to be a single 5′-end nucleotide residue A, A, G, and G located 26 bp, 24 bp, 33 bp and 33 bp upstream of the start codon according to the sequences of 20 random clones, respectively (Figs 3A, 4A, 5A and 6A). Bioinformatics analysis predicted strong SigK-like consensus binding sequences upstream of the respective start codons of all four genes (Figs 3A, 4A, 5A and 6A). The β-galactosidase assay showed that the transcriptional activities of P*bxpB* and P*cotB* were abolished in HD(Δ*sigK*) and decreased in HD(Δ*gerE*) compared with those of wild-type strain HD73 (Figs 3B and 4B). The transcriptional activity of P*bxpA* was also abolished in HD(Δ*sigK*), whereas it was increased in HD(Δ*gerE*) compared with HD73 (Fig. 5B). EMSA showed that GerE could bind to the promoters of *bxpB, cotB*, and *bxpA* (Figs 3C, 4C and 5C). To precisely determine the GerE-binding site in the *bxpB, cotB*, and *bxpA* promoters, DNase I footprinting assays were carried out using the same promoter fragments used in the EMSA. A 37-bp, 23-bp and 31-bp fragments located on *bxpB, cotB*, and *bxpA* promoters were protected by GerE binding (Figs 3D, 4D and 5D) (corresponding to the boxed sequence in the *bxpB, cotB*, and *bxpA* regions shown in Figs 3A, 4A and 5A). The transcriptional activity of P*exsY* was sharply decreased in HD(Δ*sigK*) but showed no significant difference in HD(Δ*gerE*) (Fig.6B). These results indicated that transcription of P*bxpA*, P*bxpB*, P*cotB*, and P*exsY* is controlled by SigK in the late stage of sporulation and that P*bxpA*, P*bxpB*, and P*cotB* are directly regulated by GerE.

### Transcriptional activity of the inosine hydrolase gene

**Figure 7:**
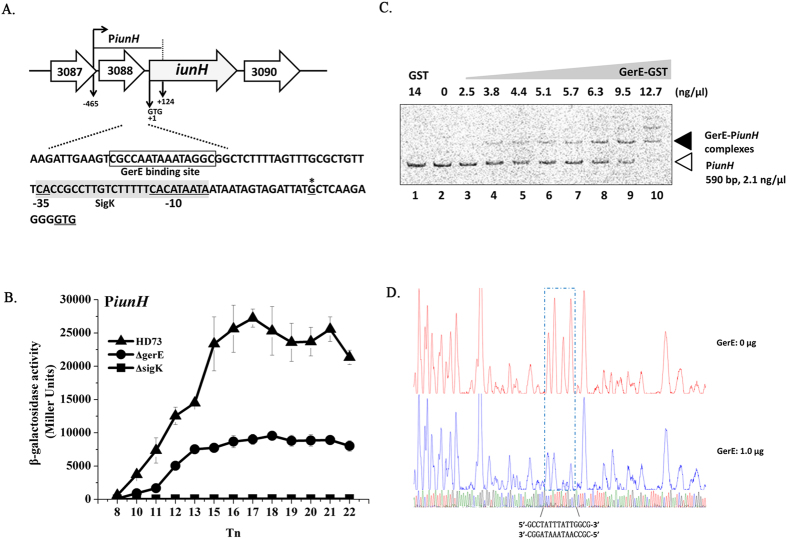
Analysis of P*iunH* transcription. (**A**) Nucleotide sequence analysis. The indicated promoter region, 465 bp upstream and 124 bp downstream of the start codon (double-underlined), was fused with *lacZ*. Transcriptional start site (TSS, indicated by asterisks) is located 10 bp upstream from the start codon of the *iunH* gene. The SigK consensus sequence is indicated, and the putative −35 and −10 sequences are underlined with a gray box. The GerE binding site is boxed. (**B**) β-galactosidase activity assay of P*iunH* in wild-type HD73 (▲), *sigK* mutant (◼), and *gerE* mutant (●). T_0_ is the end of the exponential phase, and T_n_ is n hours after T_0_. Values represent mean of at least three independent replicates; error bars represent standard deviation. (**C**) Electrophoresis mobility shift assay of the *iunH* promoter fragment (590 bp) after interaction with GerE. Lane 1, FAM-labeled P*iunH* probe incubated with GST protein; lane 2, FAM-labeled P*iunH* probe; lanes 3–10, incubation of the probe with increasing concentrations of purified GerE indicated at the top of the figure. (**D**) Protection of a 15-bp sequence in the *iunH* promoter by GerE, as revealed by DNase I footprinting protection assay. The fluorograms correspond to the DNA in the protection reactions (with 0 and 1 μg GerE).

Inosine hydrolase is encoded by *iunH* (HD73_3089) in Bt HD73, which has 93.1% identity to the homologous gene *bas2693* in the *B. anthracis* Ames strain^15^. According to the sequences of 20 random clones, the TSSs of *iunH* was confirmed to be a single 5′-end nucleotide residue G residue located 10 bp upstream of the start codon (Fig. 7A). SigK consensus binding site was present upstream of *iunH* (Fig. 7A). The β-galactosidase assay showed that the transcriptional activity of P*iunH* was abolished from T_8_ to T_22_ in HD(Δ*sigK*) and lower in HD(Δ*gerE*) than in HD73 (Fig. 7B). EMSA showed that GerE could bind to the *iunH* promoter (Fig. 7C) and DNase I footprinting assays showed that a 15-bp fragment was protected by GerE binding (Fig. 7D) (corresponding to the boxed sequence in the *iunH* region shown in Fig. 7A), together suggesting that transcription of *iunH* is controlled by SigK and is directly regulated by GerE.

## Discussion

In a *B. subtilis* mother cell, a regulatory network with a cascade of four transcription factors (SigE, SpoIIID, SigK, and GerE) controls gene expression in the mother cell during sporulation^36^. SigE and SigK are sigma subunits of RNA polymerase. SpoIIID and GerE, two small DNA-binding proteins, repress or activate transcription of many mother cell genes^31^,^37^. SigK directs the expression of most genes encoding coat structural components and factors required for spore germination, and mother-cell lysis^38^. The decisive role of SigK in spore coat assembly is evidenced by the large number of genes encoding coat structural components found in the SigK regulon^4^,^38^. Unlike the coat that constitutes the outermost layer of the mature *B. subtilis* spore^6^, the *B. cereus* group species are encircled by the exosporium^5^. Little is known about the transcription and regulation of the expression of exosporium genes in the *B. cereus* group. Indeed, only *exsB* is known to undergo SigK-mediated transcription and is positively regulated by GerE, as shown in our pervious study^39^. In this study, we first confirmed that the transcription of exosporium-related genes *bclA, bclB, bxpA, bxpB, cotB, exsY*, and *iunH* are controlled by SigK using a β-galactosidase assay. The SigK consensus sequence is located upstream of these and ten other exosporium-related genes in Bt and is predicted to be present in most *B. cereus* group strains (Additional file 2). This finding suggested that the transcription mechanisms of exosporium genes are similar throughout the *B. cereus* group.

In the *B. subtilis* cascade, the synthesis of each factor depends upon the activity of the prior factor, and there is a feedback loop in which SigK RNAP transcribes *gerE*, which then negatively regulates transcription of the *sigK* gene^31^,^40^. Some SigK-dependent genes such as oxalate decarboxylase encoded gene *oxdD*^41^ and the germination gene *gerT *30 are negatively regulated by GerE. In contrast, other SigK-dependent genes encoding spore coat proteins such as *cotB*^31^, *cotC*^31^, *yxeE*^42^, and *yeeK*^43^ are positively regulated by GerE in *B. subtilis*. We observed similar effects under the current conditions. The transcription of *bclA* and *bxpA* is negatively regulated by GerE, which could bind to the promoter of *bxpA*. Furthermore, the transcription of *bclB, bxpB, cotB*, and *iunH* is positively regulated by GerE, and their promoters could bind to GerE.

The collagen-like glycoproteins BclA and BclB require BxpB to assemble the hair-like nap of exosporium, and the assembly timing of the three proteins is similar^19^. Based on transcriptional level, we demonstrated that transcription of these three genes occurs nearly at the same stage (T_10_). BxpA is located below the spore coat associated with the cortex and is synthesized during sporulation and assembled into the spore before mother cell lysis, but it is not found in vegetative cells in *B. anthracis* Ames^44^. Furthermore, the SigK consensus sequence is found upstream of *bxpA*^13^. We provide new evidence that transcription of *bxpA* initiates at T_8_ and is abolished in the *sigK* mutant. ExsY is a homologue of *B. subtilis* cysteine-rich spore coat proteins CotY and CotZ^45^, that participates in assembly of an intact exosporium^21^. The time of synthesis of ExsY protein in the sporulation phase was detected by western-blot^21^. We confirmed that transcription of *exsY* begins at T_7_ under the control of SigK and is similar to the transcriptional mechanism of *cotYZ* in *B. subtilis*^46^. CotB is similar to ExsY in *B. anthracis*^47^ and has 30% amino acid identity to *B. subtilis* spore coat protein CotB^48^. We confirmed that the transcription of *cotB* begins at T_10_ under the control of SigK, and is regulated by GerE in Bt. The manner of transcription and regulation is similar between Bt and *B. subtilis*^31^,^36^. The transcriptional pattern of *bclA, bxpB, cotB, bxpA, exsY*, and *iunH* in wild-type HD73 is very similar, increasing from T_8_ to T_17_ and decreasing thereafter, suggesting that these proteins are assembled into the basal layer and hair-like nap simultaneously and are nearly complete at T_17_. However, the transcription of *bclB* is significantly higher than that of *blcA* after T_17_ with continuous transcriptional activity from T_8_ to T_23_. These transcriptional data are differ to previous reports, which have suggested that *bclB* and *bclA* are transcribed at an identical stage in sporulation, but with *bclB* transcribed at an approximately two-fold lower level^9^,^49^. The present data provide evidence that transcription of some exosporium genes is controlled by SigK and partially regulated by GerE. These findings provide insight into the exosporium assembly process at the transcriptional level.

## Methods

### Bacterial strains, plasmids, and growth conditions

The bacterial strains and plasmids used in this study are listed in Table 2. Bt strain HD73 was used throughout the study (accession numbers CP004069)^50^. *Escherichia coli* strain TG1 was used as the host for cloning experiments. The Dam^-^/Dcm^-^ *E. coli* ET12567 strain (laboratory stock) was used to generate unmethylated DNA for the electrotransformation assay. Bt strains were transformed by electroporation, as described previously^51^,^52^. *E. coli* and Bt strains were cultured in Luria-Bertani (LB) medium, with 220 rpm shaking at 37 °C and 30 °C, respectively. The antibiotic concentrations used for bacterial selection were as follows: 100 μg/ml kanamycin and 10 μg/ml erythromycin for Bt, and 100 μg/ml ampicillin for *E. coli*.

### DNA manipulation techniques

PCR was performed using *Taq* and KOD DNA polymerase (New England BioLabs Ltd., Beijing, China). Amplified fragments were purified using purification kits (Axygen, Union City, CA, USA). Bt chromosomal DNA was extracted with the Puregene kit (Gentra, Minneapolis, MN, USA). Restriction enzymes and T4 DNA ligase (TaKaRa Biotechnology, Dalian, China) were used according to the manufacturer’s instructions. Oligonucleotide primers (Table 3) were synthesized by Sangon (Shanghai, China). *E. coli* plasmid DNA was extracted using the Axygen Plasmid Extraction Kit. All constructs were confirmed by DNA sequencing (BGI, Beijing, China).

### Total RNA isolation and 5′-RACE analysis

For total RNA purification, strain HD73 was grown as previously described in SSM medium until the T14 stage of stationary phase (corresponding to 14 h after the end of the exponential phase)^53^. cDNA synthesis and transcriptional start sites (TSSs) of the exosporium genes were determined using the SMARTer^TM^ RACE cDNA Amplification Kit (Clontech, Mountain View, CA, USA) according to the manufacturer’s instructions. Gene-specific primers and the universal primer mix (UPM) (Table 3) were used to amplify the 5′ end of exosporium genes mRNA.

### Expression and purification of GerE

GerE protein with a glutathione *S*-transferase (GST) tag was purified from *E. coli*^54^. The *E. coli* BL21(DE3) strain carrying pGEX*gerE* plasmid was incubated in LB medium. When the optical density at 600 nm (OD600) reached 0.6, IPTG was added to a final concentration of 1 mM. After 4 h of induction at 37 °C, the bacterial cells were harvested by centrifuging the culture at 13,000 × g for 10 min. The pellet was resuspended in phosphate-buffered saline (PBS) and sonicated on ice. All subsequent procedures were carried out at 4 °C. The supernatant was collected by centrifuging the lysate at 13,000 × g for 20 min and loading it onto a glutathione-Sepharose 4B column previously equilibrated with PBS buffer. The column was washed with 50 mM Tris-HCl containing 10 mM reduced glutathione (pH 8.0). The fractions were analyzed by SDS-PAGE. Fractions with the target protein were pooled and dialyzed against PBS buffer. The purified GST-GerE protein was analyzed by SDS-PAGE on a 12% polyacrylamide gel with a protein molecular standard. All the steps described above were performed according to the manufacturer’s instructions (Amersham Pharmacia Biotech, Little Chalfont, Bucks, UK).

### Gel mobility shift assays

The DNA fragment was obtained by PCR of strain HD73 genomic DNA using specific primers (Table 3) labeled with a fluorescent 5′-end 6-FAM modification and confirmed by DNA sequencing. Electrophoresis mobility shift assays (EMSA) were performed as previously described^55^ to analyze the binding of purified GerE protein to the promoter of exosporium genes. Briefly, the DNA probe (0.1 μg) was incubated with different concentrations of purified GerE at 25 °C for 20 min in binding buffer [10 mM Tris-HCl, 0.5 mM dithiothreitol (DTT), 50 mM NaCl, 500 ng poly(dI:dC), pH 7.5, and 4% (v/v) glycerol] in a total volume of 20 μl. The DNA-protein mixtures were applied to non-denaturing 5% (w/v) polyacrylamide gels in TBE buffer (90 mM Tris-base, 90 mM boric acid, 2 mM EDTA, pH 8.0) for resolution of the complexes using a Mini-PROTEAN system (Bio-Rad) at 160 V for 1 h. Signals were visualized directly from the gel with the FLA Imager FLA-5100 (Fujifilm). The specificity of the shift was confirmed using poly(dI:dC), GST protein, and bovine serum albumin (BSA); the *cry1Ac* promoter (which does not bind to GerE protein; data not shown) was used as the negative control.

### DNase I footprinting assays

DNase I footprinting assays were performed based on a fluorescence labeling procedure^56^. Briefly, the promoters DNA of exosporium genes were PCR-amplified using the fluorescently labeled primers and purified from an agarose gel. The labeled DNA probe (400 ng) was incubated for 30 min at 25 °C with the different amounts of GerE in a total volume of 40 μl binding buffer (described above for EMSA). DNase I digestion was then performed for 1 min at 25 °C and stopped with stop buffer (Promega). After phenol-chloroform extraction and ethanol precipitation, the samples were loaded on an Applied Biosystems 3730 DNA genetic analyzer with an internal-lane size standard (ROX-500, Applied Biosystems). A dye primer-based sequencing kit (Thermo) was used to precisely determine the sequences after their alignment wtih capillary electrophoresis results. Electropherograms were analyzed with GeneMarker v1.8 (Applied Biosystems).

### Construction of the promoters of exosporium genes with *lacZ* gene fusion

The promoters of exosporium genes were amplified from Bt HD73 genomic DNA using specific primers. Promoter restriction fragments were then ligated into the pHT304-18Z vector containing a promoterless *lacZ* gene^57^. Recombinant pHT-Pn (where n indicates the name of exosporium genes) was introduced into Bt HD73, *ΔsigK* and *ΔgerE* mutant strains. The resultant strains, HD73(Pn), *ΔsigK*(Pn), and *ΔgerE*(Pn), were selected by resistance to erythromycin and tested by PCR to confirm the presence of the promoter fragments in the plasmids.

### β-Galactosidase assays

Bt strains containing *lacZ* transcriptional fusions were cultured in Schaeffer’s sporulation medium (SSM)^58^ at 30 °C and 220 rpm. A 2-ml volume was collected at 1-h intervals from T_8_ to T_22_ (T_0_ is the end of the exponential phase, and T_n_ is n hours after T_0_), from which cells were harvested by centrifugation for 1 min at 10,000 × *g*. The supernatant was removed, and the pellet was stored at −20 °C or resuspended in 500 μl Buffer Z (0.06 M Na_2_HPO_4_, 0.04 M NaH_2_PO_4_, 0.01 M KCl, 1 mM MgSO_4_) with 1 mM dithiothreitol. The β-galactosidase activity was determined as previously described^59^ and expressed as Miller units. Reported values represent averages from at least three independent assays.

## Additional Information

**How to cite this article**: Peng, Q. *et al.* The Regulation of Exosporium-Related Genes in *Bacillus thuringiensis. Sci. Rep.* **6**, 19005; doi: 10.1038/srep19005 (2016).

## Supplementary Material

Supplementary Information

## Figures and Tables

**Table 1:** Exosporium homologous genes in Bt HD73.

**Gene ID**	**Gene name**	**Source of homologous gene**	**Identity**	**Structure**	**Ref.**
HD73_1438	*bclA*	*B. anthracis* Sterne strain 7702	67.8%	Hairy nap	33
HD73_2664	*bclB*	*B. cereus* ATCC 10876	90.0%	unknown	34,60
HD73_2410	*bxpA*	*B. anthracis*	75.4%	Basal layer	13
HD73_1452	*bxpB*	*B. anthracis* Ames	97.0%	Basal layer	15
HD73_0469	*cotB*	*B. anthracis* Ames	76.9%	Basal layer	15
HD73_1453	*cotY*	*B. cereus* ATCC 10876	92.9%	Basal layer	21
HD73_2208	*exsB*	*B. anthracis* Sterne	92.0%	Basal layer	23
HD73_1449	*exsY*	*B. cereus* ATCC 10876	87.0%	Basal layer	21
HD73_3089	*iunH*	*B. anthracis* Ames	93.1%	Enzyme	15
HD73_4056	*cotE*	*B. cereus* ATCC 10876	100%	Basal layer	34
HD73_4735	*exsA*	*B. cereus* ATCC 10876	89.4%	Basal layer	22
HD73_3082	*exsC*	*B. cereus* ATCC 10876	93.8%	Basal layer	34
HD73_1094	*exsD*	*B. cereus* ATCC 10876	46.2%	Basal layer	34
HD73_1970	*exsE*	*B. cereus* ATCC 10876	99.6%	Basal layer	34
HD73_2393	*exsG*	*B. cereus* ATCC 10876	100%	Basal layer	34
HD73_3464	*exsK*	*B. anthracis* Ames	64.4%	Basal layer	24
HD73_5608	*exsM*	*B. cereus* ATCC 14579	100%	Basal layer	25

**Table 2:** Strains and plasmids.

**Strain or plasmid**	**Relevant genotype and characteristics**^**a**^	**Reference or source**
Strains		
HD73	Bt subsp. *Kurstaki* carrying the *cry1Ac* gene	Laboratory collection
HD(Δ*sigK*)	Bt HD73 *sigK* gene mutant; Kan^R^	54
HD(Δ*gerE*)	Bt HD73 *gerE* gene mutant	54
HD(P*bxpA*)	Bt HD73 carrying pHT-PbxpA plasmid; Em^R^	This study
HD(P*bxpB*)	Bt HD73 carrying pHT-PbxpB plasmid; Em^R^	This study
HD(P*bclA*)	Bt HD73 carrying pHT-PbclA plasmid; Em^R^	This study
HD(P*bclB*)	Bt HD73 carrying pHT-PbclB plasmid; Em^R^	This study
HD(P*cotB*)	Bt HD73 carrying pHT-PcotB plasmid; Em^R^	This study
HD(P*exsY*)	Bt HD73 carrying pHT-PexsY plasmid; Em^R^	This study
HD(P*iunH*)	Bt HD73 carrying pHT-PiunH plasmid; Em^R^	This study
Δ*sigK*(P*bxpA*)	HD(Δ*sigK*) carrying pHT-PbxpA plasmid; Em^R^	This study
Δ*sigK* (P*bxpB*)	HD(Δ*sigK*) carrying pHT-PbxpB plasmid; Em^R^	This study
Δ*sigK* (P*bclA*)	HD(Δ*sigK*) carrying pHT-PbclA plasmid; Em^R^	This study
Δ*sigK* (P*bclB*)	HD(Δ*sigK*) carrying pHT-PbclB plasmid; Em^R^	This study
Δ*sigK* (P*cotB*)	HD(Δ*sigK*) carrying pHT-PcotB plasmid; Em^R^	This study
Δ*sigK* (P*exsY*)	HD(Δ*sigK*) carrying pHT-PexsY plasmid; Em^R^	This study
Δ*sigK* (P*iunH*)	HD(Δ*sigK*) carrying pHT-PiunH plasmid; Em^R^	This study
Δ*gerE*(P*bxpA*)	HD(Δ*gerE*) carrying pHT-PbxpA plasmid; Em^R^	This study
Δ*gerE* (P*bxpB*)	HD(Δ*gerE*) carrying pHT-PbxpB plasmid; Em^R^	This study
Δ*gerE* (P*bclA*)	HD(Δ*gerE*) carrying pHT-PbclA plasmid; Em^R^	This study
Δ*gerE* (P*bclB*)	HD(Δ*gerE*) carrying pHT-PbclB plasmid; Em^R^	This study
Δ*gerE* (P*cotB*)	HD(Δ*gerE*) carrying pHT-PcotB plasmid; Em^R^	This study
Δ*gerE* (P*exsY*)	HD(Δ*gerE*) carrying pHT-PexsY plasmid; Em^R^	This study
Δ*gerE* (P*iunH*)	HD(Δ*gerE*) carrying pHT-PiunH plasmid; Em^R^	This study
*E. coli* TG1	Δ(*lac-proAB*) *supE thi hsd-*5 (*F’ traD36 proA*^+^ *proB*^+^ *lacI*^q^ *lacZ*ΔM15), general purpose cloning host	Laboratory collection
*E. coli* ET12567	*F*^-^ *dam-13*::Tn*9 dcm-6 hsdM hsdR recF143 zjj-202*::Tn*10 galK2 galT22 ara14 pacY1 xyl-5 leuB6 thi-1*, for generation of unmethylated DNA	Laboratory collection
BL (pGEX*gerE*)	BL21(DE3) with pGEX*gerE* plasmid	54
Plasmids		
pHT304-18Z	Promoterless *lacZ* vector, Em^R^, Ap^R^	Laboratory collection
pHT-PbxpA	pHT304-18Z carrying promoter upstream from *bxpA*	This study
pHT-PbxpB	pHT304-18Z carrying promoter upstream from *bxpB*	This study
pHT-PbclA	pHT304-18Z carrying promoter upstream from *bclA*	This study
pHT-PbclB	pHT304-18Z carrying promoter upstream from *bclB*	This study
pHT-PcotB	pHT304-18Z carrying promoter upstream from *cotB*	This study
pHT-PexsY	pHT304-18Z carrying promoter upstream from *exsY*	This study
pHT-PiunH	pHT304-18Z carrying promoter upstream from *iunH*	This study

**Table 3:** Primers sequences.

**oligonucleotides**	**sequence (5′-3′)**^a^
PbxpA-F	AACTGCAGATAAGACATATTGGCGATGA
PbxpA-R	CGGGATCCTTTCTTGATTTTGCGTTG
PbxpB-F	AACTGCAGGCATTTGCACCATCTTCA
PbxpB-R	CGGGATCCTTGGGTTTGGACTTACGC
PbclA-F	AACTGCAGCTCCTTGCGTCGCTTTTGA
PbclA-R	CGGGATCCCGGTGGTATCGGTGGTAA
PbclB-F	AACTGCAGATGGTTGAATGATAGGCA
PbclB-R	CGGGATCCATCGGAACTGTTTGTGGA
PcotB-F	AACTGCAGAAAATTCGTGCGCTATTC
PcotB-R	CGGGATCCCTGCTTTACAATCTTTCG
PexsY-F	CCCAAGCTTCGGTTCCGCAACGATAGG
PexsY-R	AACTGCAGGGGCGTGTATTTGCTACTGAT
PiunH-F	AACTGCAGGATGAAAGCACCAAACGA
PiunH-R	CGGGATCCTTCCCATACTCAGCAACAAT
PbxpB-a	AAGACTAATATCAACCTCCAC
PbxpB-b	GTAAATTCGCAATCAGAAGA
PbxpA-a	ATCCACTTTACCGCCATG
PbxpA-b	TTGATTTTGCGTTGTTGC
PbclB-a	TGTTAATCGTAAATTCGG
PbclB-b	ATTGCAGTGGTTATGACC
PcotB-a	AAGACGAAGATTAAACTATG
PcotB-b	AACTCACGAGAAAACCC
PiunH-a	GATGAAAGCACCAAACGA
PiunH-b	TTCCCATACTCAGCAACAAT
bxpARACE	GCGTTGTTGCATATGGG
bxpBRACE	TTGGGTTTGGACTTACGCTAG
bclARACE	CGGTGGTATCGGTGGTAATG
bclBRACE	ATCGGAACTGTTTGTGGATTG
cotBRACE	CTTCAACTTTCTCTGGGCCA CCACGA
exsYRACE	CGGCAGCTAGTAAGGCTTGAAGATGGTG
iunHRACE	CCGTAACGATATCTCGTG
UPM	AAGCAGTGGTATCAACGCAGAGTACATGGG

^a^Restriction enzyme sites are underscored.
