# Supplementary material for: The Regulation of Exosporium-Related Genes in Bacillus thuringiensis
Source: Sci Rep. 2016 Jan 25;6:19005. doi: 10.1038/srep19005 (PMC4750369; doi:10.1038/srep19005)
Supplement: Supplementary Information [file srep19005-s1.pdf]

The Regulation of Exosporium-Related Genes in *Bacillus thuringiensis*

Qi Peng[1], Guiwei Kao[1], Ning Qu[1, 2], Jie Zhang[1], Jie Li[2], Fuping Song[1*]

[1]State Key Laboratory for Biology of Plant Diseases and Insect Pests, Institute of Plant Protection,

Chinese Academy of Agricultural Sciences, Beijing

[2]College of Life Sciences, Northeast Agriculture University, Harbin, China

[*]Address correspondence to Fuping Song, fpsong@ippcaas.cn

Additional file 1. Electrophoresis mobility shift assay of the *bclA* (A) *and exsY* (B) promoters with GerE

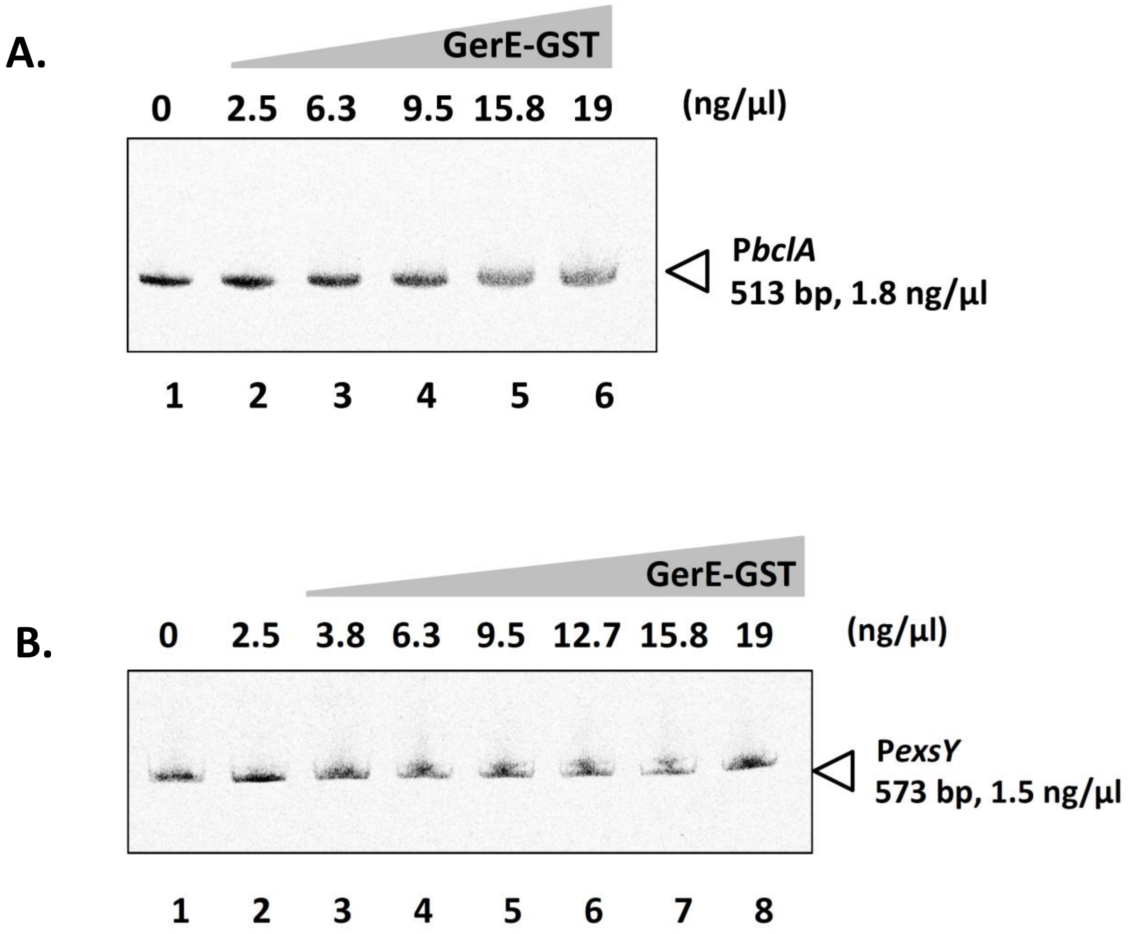

A. lane 1, FAM-labeled P*bclA* probe; lanes 2–6, incubation of the probe with increasing concentrations of purified GerE indicated at the top of the figure. B. lane 1, FAM-labeled P*exsY* probe; lanes 2–8, incubation of the probe with increasing concentrations of purified GerE indicated at the top of the figure.

Additional file 2: SigK consensus sequence of exosporium genes in *B. cereus* group strains

		−35 −10
	Sigma K Consensus	MWCM----space=16----CATANNNTD*
bclA	*B. thuringiensis* HD73	CACCCTCTTCCAAATCAAT-CATATGTTA
	B. cereus ATCC 14579	CACCCTCTTCCAAATCAAT-CATATGTTA
	B. cereus B4264	CACCCTCTTCCAAATCAAT-CATATGTTA
	B. cereus G9842	CACCCTCTTCCAAATCAAT-CATATGTTA
	B. thuringiensis 97-27	CACCCTCTTCCAAATCAAT-CATATGTTA
	B. thuringiensis BMB171	CACCCTCTTCCAAATCAAT-CATATGTTA
	B. thuringiensis 407	CACCCTCTTCCAAATCAAT-CATATGTTA
	B. thuringiensis CT-43	CACCCTCTTCCAAATCAAT-CATATGTTA
	B. thuringiensis HD-789	CACCCTCTTCCAAATCAAT-CATATGTTA
	B. thuringiensis HD-771	CACCCTCTTCCAAATCAAT-CATATGTTA
	B. weihenstephanensis KBAB4	CACCCTCTTCCAAATCAAT-CATATGTTA
	B. anthracis str. Ames	CACCCTCTTCCAAATCAAT-CATATGTTA
	B. anthracis str. Sterne	CACCCTCTTCCAAATCAAT-CATATGTTA

* D is A, G, or T; N is A, C, G, or T; M is A or C; W is A or T.

		−35 −10
	Sigma K Consensus	MWCM----space=16----CATANNNTD*
bclB	*B. thuringiensis* HD73	AGCAGTCAATTATTAAAAA-CATATAATT
	B. cereus ATCC 14579	AGCAGTCAATTATTAAAAA-CATATAATT
	B. cereus B4264	AGCAGTCAATTATTAAAAA-CATATAATT
	B. cereus G9842	AGCAGTCAATTATTAAAAA-CATATAATT
	B. thuringiensis 97-27	AGCAGTCAATTATTAAAAA-CATATAATT
	B. thuringiensis BMB171	AGCAGTCAATTATTAAAAA-CATATAATT
	B. thuringiensis 407	AGCAGTCAATTATTAAAAA-CATATAATT
	B. thuringiensis CT-43	AGCAGTCAATTATTAAAAA-CATATAATT
	B. thuringiensis HD-789	AGCAGTCAATTATTAAAAA-CATATAATT
	B. thuringiensis HD-771	AGCAGTCAATTATTAAAAA-CATATAATT
	B. weihenstephanensis KBAB4	AGCAGTCAATTATTAAAAA-CATATAATT
	B. anthracis str. Ames	AGCAGTCAATTATTAAAAA-CATATAATT
	B. anthracis str. Sterne	AGCAGTCAATTATTAAAAA-CATATAATT

		−35 −10
	Sigma K Consensus	MWCM----space=16----CATANNNTD*
bxpA	*B. thuringiensis* HD73	CACATATTTTCTTGTCCGCCCATACACTA
	B. cereus ATCC 14579	CACATATTTTCTTGTCCGCCCATACACTA
	B. cereus B4264	CACATATTTTCTTGTCCGCCCATACACTA
	B. cereus G9842	CACATATTTTCTTGTCCGCCCATACACTA
	B. thuringiensis 97-27	CACATATTTTCTTGTCCGCCCATACACTA
	B. thuringiensis BMB171	CACATATTTTCTTGTCCGCCCATACACTA
	B. thuringiensis 407	CACATATTTTCTTGTCCGCCCATACACTA
	B. thuringiensis CT-43	CACATATTTTCTTGTCCGCCCATACACTA
	B. thuringiensis HD-789	CACATATTTTCTTGTCCGCCCATACACTA
	B. thuringiensis HD-771	CACATATTTTCTTGTCCGCCCATACACTA
	B. weihenstephanensis KBAB4	CACATATTTTCTTGTCCGCCCATACACTA
	B. anthracis str. Ames	CACATATTTTCTTGTCCGCCCATACACTA
	B. anthracis str. Sterne	CACATATTTTCTTGTCCGCCCATACACTA

		−35 −10
	Sigma K Consensus	MWCM----space=16----CATANNNTD*
bxpB	*B. thuringiensis* HD73	AACATAGAACCTGTCCTTTTCATTAACTG
	B. cereus ATCC 14579	AACATAGAACCTGTCCTTTTCATTAACTG
	B. cereus B4264	AACATAGAACCTGTCCTTTTCATTAACTG
	B. cereus G9842	AACATAGAACCTGTCCTTTTCATTAACTG
	B. thuringiensis 97-27	AACATAGAACCTGTCCTTTTCATTAACTG
	B. thuringiensis BMB171	AACATAGAACCTGTCCTTTTCATTAACTG
	B. thuringiensis 407	AACATAGAACCTGTCCTTTTCATTAACTG
	B. thuringiensis CT-43	AACATAGAACCTGTCCTTTTCATTAACTG
	B. thuringiensis HD-789	AACATAGAACCTGTCCTTTTCATTAACTG
	B. thuringiensis HD-771	AACATAGAACCTGTCCTTTTCATTAACTG
	B. weihenstephanensis KBAB4	AACATAGAACCTGTCCTTTTCATTAACTG
	B. anthracis str. Ames	AACATAGAACCTGTCCTTTTCATTAACTG
	B. anthracis str. Sterne	AACATAGAACCTGTCCTTTTCATTAACTG

		-35 ... -10
	Sigma K Consensus	MWCM----space=16----CATANNNTD*
cotB	*B. thuringiensis* HD73	-AACGTAACCTGTACAGATACATATACTA
	B. cereus ATCC 14579	-AACGTAACCTGTACAGATACATATACTA
	B. cereus B4264	-AACGTAACCTGTACAGATACATATACTA
	B. cereus G9842	-AACGTAACCTGTACAGATACATATACTA
	B. thuringiensis 97-27	-AACGTAACCTGTACAGATACATATACTA
	B. thuringiensis BMB171	-AACGTAACCTGTACAGATACATATACTA
	B. thuringiensis 407	-AACGTAACCTGTACAGATACATATACTA
	B. thuringiensis CT-43	-AACGTAACCTGTACAGATACATATACTA
	B. thuringiensis HD-789	-AACGTAACCTGTACAGATACATATACTA
	B. thuringiensis HD-771	-AACGTAACCTGTACAGATACATATACTA
	B. weihenstephanensis KBAB4	-AACGTAACCTGTACAGATACATATACTA
	B. anthracis str. Ames	-AACGTAACCTGTACAGATACATATACTA
	B. anthracis str. Sterne	-AACGTAACCTGTACAGATACATATACTA

		-35 ... -10
	Sigma K Consensus	MWCM----space=16----CATANNNTD*
cotY	*B. thuringiensis* HD73	AATATCAGCCATATCCCACTCATATTGTA
	B. cereus ATCC 14579	AATATCAGCCATATCCCACTCATATTGTA
	B. cereus B4264	AATATCAGCCATATCCCACTCATATTGTA
	B. cereus G9842	AATATCAGCCATATCCCACTCATATTGTA
	B. thuringiensis 97-27	AATATCAACCATATCTCATTCATATTGTA
	B. thuringiensis BMB171	AATATCAGCCATATTCCACTCATATTGTA
	B. thuringiensis 407	AATATCAACCATATCCCACTCATATTGTA
	B. thuringiensis CT-43	AATATCAACCATATCCCACTCATATTGTA
	B. thuringiensis HD-789	AATATCAGCCATATCCCACTCATATTGTA
	B. thuringiensis HD-771	AATATCAGCCATATCCCACTCATATTGTA
	B. weihenstephanensis KBAB4	AATATCAACCATATCCCGTTCATATTGTA
	B. anthracis str. Ames	AATATCAACCATATCCCGTTCATATTGTA
	B. anthracis str. Sterne	AATATCAACCATATCCCGTTCATATTGTA

		-35 -10
	Sigma K Consensus	MWCM----space=16----CATANNNTD*
exsY	*B. thuringiensis* HD73	-ACCAAATCTCCTCGACTCCAATATCTTA
	B. cereus ATCC 14579	-ACCAAATCTCCTCGACTCCAATATCTTA
	B. cereus B4264	-ACCAAATCTCCTCGACTCCAATATCTTA
	B. cereus G9842	-ACCAAATCTCCTCGACTCCAATATCTTA
	B. thuringiensis 97-27	-ACCAAATCTCCTCAACTCCAATATCTTA
	B. thuringiensis BMB171	-ACCAAATCTCCTCGACTCCAATATCTTA
	B. thuringiensis 407	-ACCAAATCTCCTCGACTCCAATATCTTA
	B. thuringiensis CT-43	-ACCAAATCTCCTCGACTCCAATATCTTA
	B. thuringiensis HD-789	-ACCAAATCTCCTCGACTCCAATATCTTA
	B. thuringiensis HD-771	-ACCAAATCTCCTCGACTCCAATATCTTA
	B. weihenstephanensis KBAB4	-ACCAAATCCCCTCCACTCCAATATCTTA
	B. anthracis str. Ames	-ACCAAATCTCCTCGACTCCAATATCTTA
	B. anthracis str. Sterne	-ACCAAATCTCCTCGACTCCAATATCTTA

		-35 -10
	Sigma K Consensus	MWCM----space=16----CATANNNTD*
iunh	*B. thuringiensis* HD73	--CACCGCCTTGTCTTTTT-CACATAATA
	B. cereus ATCC 14579	--CACCGCCTTGTCTTTTT-CACATAATA
	B. cereus B4264	--CACCGCCTTGTCTTTTT-CACATAATA
	B. cereus G9842	--CACCGCCTTGTCTTTTT-CACATAATA
	B. thuringiensis 97-27	--CACAGCCTTGTCTTTTT-TACATACTA
	B. thuringiensis BMB171	--CACCGCCTTGTCTTTTT-CACATAATA
	B. thuringiensis 407	--CACCGCCTTGTCTTTTT-CACATAATA
	B. thuringiensis CT-43	--CACCGCCTTGTCTTTTT-CACATAATA
	B. thuringiensis HD-789	--CACCGCCTTGTCTTTTT-CACATAATA
	B. thuringiensis HD-771	--CACCGCCTTGTCTTTTT-CACATAATA
	B. weihenstephanensis KBAB4	--CACCGCCTTGTCTTTTT-CTCATAATA
	B. anthracis str. Ames	--CACAGCCTTGTCTTTTT-TACATACTA
	B. anthracis str. Sterne	--CACAGCCTTGTCTTTTT-TACATACTA

		-35 ... -10
	Sigma K Consensus	MWCM----space=16----CATANNNTD*
cotE	*B. thuringiensis* HD73	CACATTCCGCTATTGTCACGCATATGATT
	B. cereus ATCC 14579	CACATTCCGCTATTGTCACGCATATGATT
	B. cereus B4264	CACATTCCGCTATTGTCACGCATATGATT
	B. cereus G9842	CACATTCCGCTATTGTCACGCATATGATT
	B. thuringiensis 97-27	CACATTCCGCTATTGTCACGCATATGATT
	B. thuringiensis BMB171	CACATTCCGCTATTGTCACGCATATGATT
	B. thuringiensis 407	CACATTCCGCTATTGTCACGCATATGATT
	B. thuringiensis CT-43	CACATTCCGCTATTGTCACGCATATGATT
	B. thuringiensis HD-789	CACATTCCGCTATTGTCACGCATATGATT
	B. thuringiensis HD-771	CACATTCCGCTATTGTCACGCATATGATT
	B. weihenstephanensis KBAB4	CACATTCCGCTATTGTCACGCATATGATT
	B. anthracis str. Ames	CACATTCCGCTATTGTCACGCATATGATT
	B. anthracis str. Sterne	CACATTCCGCTATTGTCACGCATATGATT

		-35 ... -10
	Sigma K Consensus	MWCM----space=16----CATANNNTD*
exsC	*B. thuringiensis* HD73	AGCTAATTCTTCACTATTTTCATATTCTA
	B. cereus ATCC 14579	AGCTAATTCTTAACTATTTTAATATTCTA
	B. cereus B4264	AGCTAATTCTTCACTATTTTCATATTCTA
	B. cereus G9842	AGATAATTCATCCCTATTTCCGTATTTCA
	B. thuringiensis 97-27	ATCTAATCCGTCATTACGTTCATATTTTA
	B. thuringiensis BMB171	AGCTAATTCTTAACTATTTTAATATTCTA
	B. thuringiensis 407	AGCTAATTCGTCACCTCCT-CATATTCTA
	B. thuringiensis CT-43	AGCTAATTCGTCACCTCCT-CATATTCTA
	B. thuringiensis HD-789	AGATAATTCATCCCTATTTCCGTATTTCA
	B. thuringiensis HD-771	AGATAATTCATCCCTATTTCCGTATTTCA
	B. weihenstephanensis KBAB4	AGCTAATTCATCGTTACCTTCATATTTTA
	B. anthracis str. Ames	ATCTAATCCGTCATTACGTTCATATTTTT
	B. anthracis str. Sterne	ATCTAATCCGTCATTACGTTCATATTTTT

		-35 -10
	Sigma K Consensus	MWCM----space=16----CATANNNTD*
exsD	*B. thuringiensis* HD73	AACCTTTTTATACAACCTT-CATAATATA
	B. cereus ATCC 14579	AACATTCACATGTTATCTT-CAATATGTT
	B. cereus B4264	AACCTTTTTATACAAGTTT-CATAATATA
	B. cereus G9842	AACATATAAAAATATATTT-CATATTATA
	B. thuringiensis 97-27	TACCTCTCTAAAAACCATG-CATACAATA
	B. thuringiensis BMB171	AACATTCACATGTTATCTT-CAATATGTT
	B. thuringiensis 407	AACATTCACATGTTATCTT-CAATATGTT
	B. thuringiensis CT-43	AACATTCACATGTTATCTT-CAATATGTT
	B. thuringiensis HD-789	AACATATAAAAATATATTT-CATATTATA
	B. thuringiensis HD-771	AACATATAAAAATATATTT-CATATTATA
	B. weihenstephanensis KBAB4	AATTTTTTCTCTTATTCTC-CTAAATAGA
	B. anthracis str. Ames	AATTTTCAAATAACATTTT-CAGGTAATT
	B. anthracis str. Sterne	AATTTTCAAATAACATTTT-CAGGTAATT

		-35 -10
	Sigma K Consensus	MWCM----space=16----CATANNNTD*
exsE	*B. thuringiensis* HD73	CACATCCTTTCTCCGTGTAACATTTCCTA
	B. cereus ATCC 14579	CACATCCTTTCTCCGTGTAACATTTCCTA
	B. cereus B4264	CACATCCTTTCTTCCCGTAACATTTCCTA
	B. cereus G9842	CACATCCTTTCTCCGTATAACATTTCCTA
	B. thuringiensis 97-27	CACATCCTTTCTCTGTGTAACATTTCCTA
	B. thuringiensis BMB171	CACATCCTTTCTCCGTGTAACATTTCCTA
	B. thuringiensis 407	CACATCCTTTCTTCCCGTAACATTTCCTA
	B. thuringiensis CT-43	CACATCCTTTCTTCCCGTAACATTTCCTA
	B. thuringiensis HD-789	CACATCCTTTCTCCGTATAACATTTCCTA
	B. thuringiensis HD-771	CACATCCTTTCTCCGTATAACATTTCCTA
	B. weihenstephanensis KBAB4	CACATCCTTTCTCCATGTAGCATTTCCTA
	B. anthracis str. Ames	CACATCCTTTCTCCGTGTAACATTTCCTA
	B. anthracis str. Sterne	CACATCCTTTCTCCGTGTAACATTTCCTA

		-35 -10
	Sigma K Consensus	MWCM----space=16----CATANNNTD*
exsG	*B. thuringiensis* HD73	TTACTTTTTGAAAGAGATTTCATAAATTA
	B. cereus ATCC 14579	TTACGTTTTGAAAGAGATTTCATAAATTA
	B. cereus B4264	TTACTTTTTGAAAGAGATTTCATAAATTA
	B. cereus G9842	TTACTTTTTGAAAGAGATTTCATAAATTA
	B. thuringiensis 97-27	-----------------------------
	B. thuringiensis BMB171	TTACGTTTTGAAAGAGATTTCATAAATTA
	B. thuringiensis 407	TTACTTTTTGAAAGATATTTCATAAATTA
	B. thuringiensis CT-43	TTACTTTTTGAAAGATATTTCATAAATTA
	B. thuringiensis HD-789	TTACTTTTTGAAAGAGATTTCATAAATTA
	B. thuringiensis HD-771	TTACTTTTTGAAAGAGATTTCATAAATTA
	B. weihenstephanensis KBAB4	TTACATTTTGAAAGAGATTTCATAAATTA
	B. anthracis str. Ames	-----------------------------
	B. anthracis str. Sterne	-----------------------------

		-35 -10
	Sigma K Consensus	MWCM----space=16----CATANNNTD*
exsK	*B. thuringiensis* HD73	TACCCTTTTAAGCTAGTGGACATACTTTA
	B. cereus ATCC 14579	TACCCTTTTAAGCTAGTGGACATACTTTA
	B. cereus B4264	TACCCTTTTAAGCTAGTGGACATACTTTA
	B. cereus G9842	TACCCTTTTAAGCTAGTGGACATAATTTA
	B. thuringiensis 97-27	TACCCTTTGAAGCTAGTGGACATACTTTA
	B. thuringiensis BMB171	TACCCTTTTAAGCTAGTGGACATACTTTA
	B. thuringiensis 407	TACCCTTTTAAGCTAGTGGACATACTTTA
	B. thuringiensis CT-43	TACCCTTTTAAGCTAGTGGACATACTTTA
	B. thuringiensis HD-789	TACCCTTTTAAGCTAGTGGACATACTTTA
	B. thuringiensis HD-771	TACCCTTTTAAGCTAGTGGACATACTTTA
	B. weihenstephanensis KBAB4	TACCCTTTTAAGCTATTGGACATACTTTA
	B. anthracis str. Ames	TACCCTTTGAAGCTAGTGGACATACTTTA
	B. anthracis str. Sterne	TACCCTTTGAAGCTAGTGGACATACTTTA

		-35 -10
	Sigma K Consensus	MWCM----space=16----CATANNNTD*
exsM	*B. thuringiensis* HD73	--AAATGATTATGTTTAAACCATAAAGTA
	B. cereus ATCC 14579	--AAATGATTATGTCTAAACCATAAAGTA
	B. cereus B4264	--AAATGATTATGTCTAAACCATAAAGTA
	B. cereus G9842	--AAATGATTATGTCTAAACCATAAAGTA
	B. thuringiensis 97-27	--AAATGATTATGTATAAACCATAAAGTA
	B. thuringiensis BMB171	--AAATGATTATGTCTAAACCATAAAGTA
	B. thuringiensis 407	--AAATGATTATGTCTAAACCATAAAGTA
	B. thuringiensis CT-43	--AAATGATTATGTCTAAACCATAAAGTA
	B. thuringiensis HD-789	--AAATGATTATGTCTAAACCATAAAGTA
	B. thuringiensis HD-771	--AAATGATTATGTCTAAACCATAAAGTA
	B. weihenstephanensis KBAB4	--AAATGATTATGTCTAAACCATAAAGTA
	B. anthracis str. Ames	--AAATGATTATGTATAAACCATAAAGTA
	B. anthracis str. Sterne	--AAATGATTATGTATAAACCATAAAGTA
